# Supplementary material for: An experimental target-based platform in yeast for screening Plasmodium vivax deoxyhypusine synthase inhibitors
Source: PLoS Negl Trop Dis. 2024 Dec 2;18(12):e0012690. doi: 10.1371/journal.pntd.0012690 (PMC11637365; doi:10.1371/journal.pntd.0012690)
Supplement: S1 Fig — (A) Cartoon representation of the PvDHS—model 1 (red) superposed on the HsDHS structure 6P4V [32] (blue). (B) Overlay of PvDHS—model 2 (yellow) superposed on the HsDHS structure 6PGR [32] (magenta). (C) Overlay of α-helix unfolded in PvDHS-model 2 (gray) with the corresponding position in PvDHS-model1 (green). (DOCX) [file pntd.0012690.s001.docx]

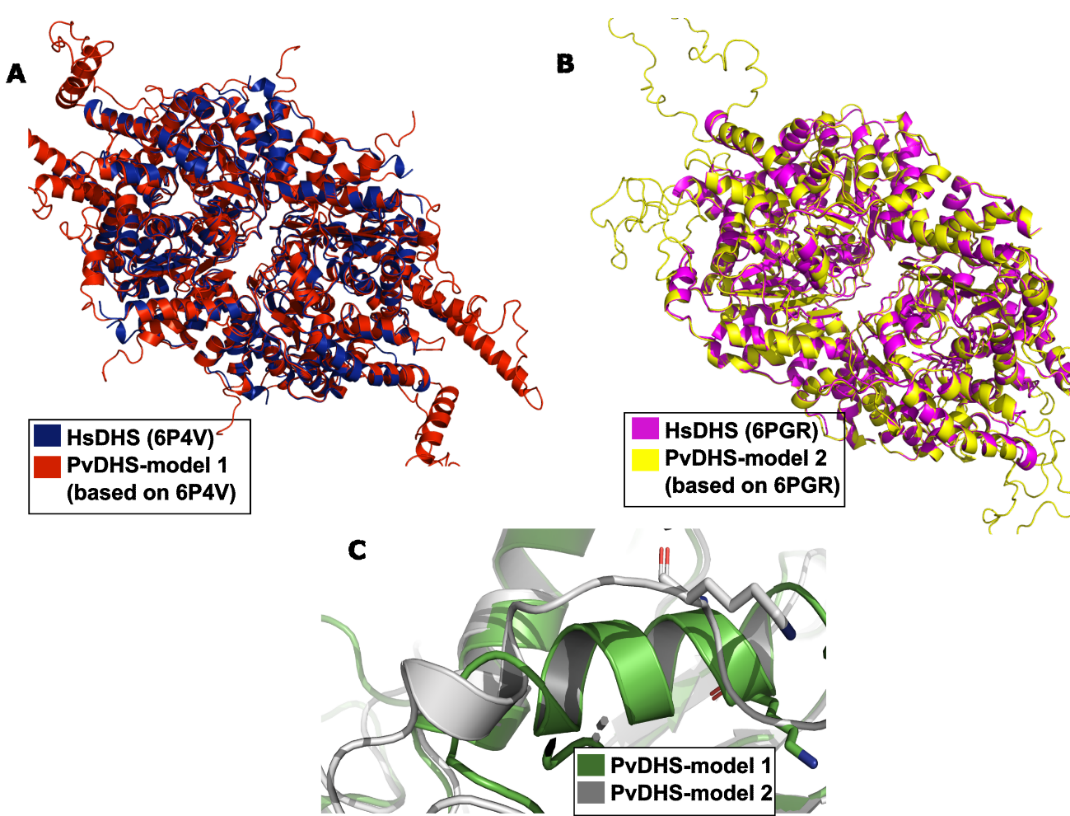


**S1 Fig.** Homology models of *P. vivax* DHS homotetramer.

(A) Cartoon representation of the PvDHS - model 1 (red) superposed on the HsDHS structure 6P4V [32] (blue). (B) Overlay of PvDHS - model 2 (yellow) superposed on the HsDHS structure 6PGR [32] (magenta). (C) Overlay of α-helix unfolded in PvDHS-model 2 (gray) with the corresponding position in PvDHS-model1 (green).
