# Supplementary material for: An experimental target-based platform in yeast for screening Plasmodium vivax deoxyhypusine synthase inhibitors
Source: PLoS Negl Trop Dis. 2024 Dec 2;18(12):e0012690. doi: 10.1371/journal.pntd.0012690 (PMC11637365; doi:10.1371/journal.pntd.0012690)
Supplement: S2 Fig — The black dashed line indicates the threshold distinguishing poor- and high-quality local similarity regions. Arrows indicate the position of the residues within the binding sites. (DOCX) [file pntd.0012690.s002.docx]

**
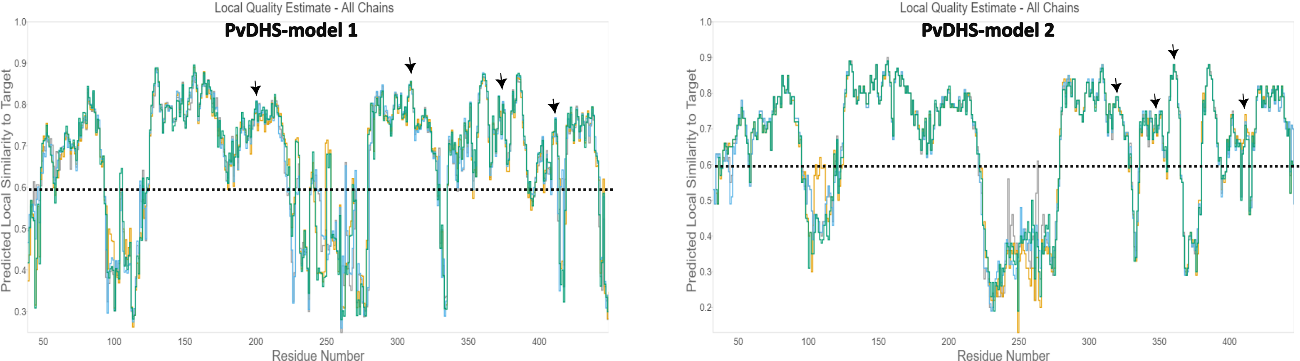
**

**S2 Fig.** Local quality assessment of residues in the PvDHS-model 1 and

PvDHS-model 2.

The black dashed line indicates the threshold distinguishing poor- and high-quality local similarity regions. Arrows indicate the position of the residues within the binding sites.
