# Supplementary material for: An experimental target-based platform in yeast for screening Plasmodium vivax deoxyhypusine synthase inhibitors
Source: PLoS Negl Trop Dis. 2024 Dec 2;18(12):e0012690. doi: 10.1371/journal.pntd.0012690 (PMC11637365; doi:10.1371/journal.pntd.0012690)
Supplement: S3 Fig — Zoomed-out view of GC7 (pink) and 8XY (red) disposition in the tetramer structures 6P4V (A) and PDB ID: 6PGR (C), respectively. B and D show the overlay of the original ligands, GC7 (pink) and 8XY (red) with the ligands after redocking (gray). Different colors represent individual chains in the structure. (DOCX) [file pntd.0012690.s003.docx]

**
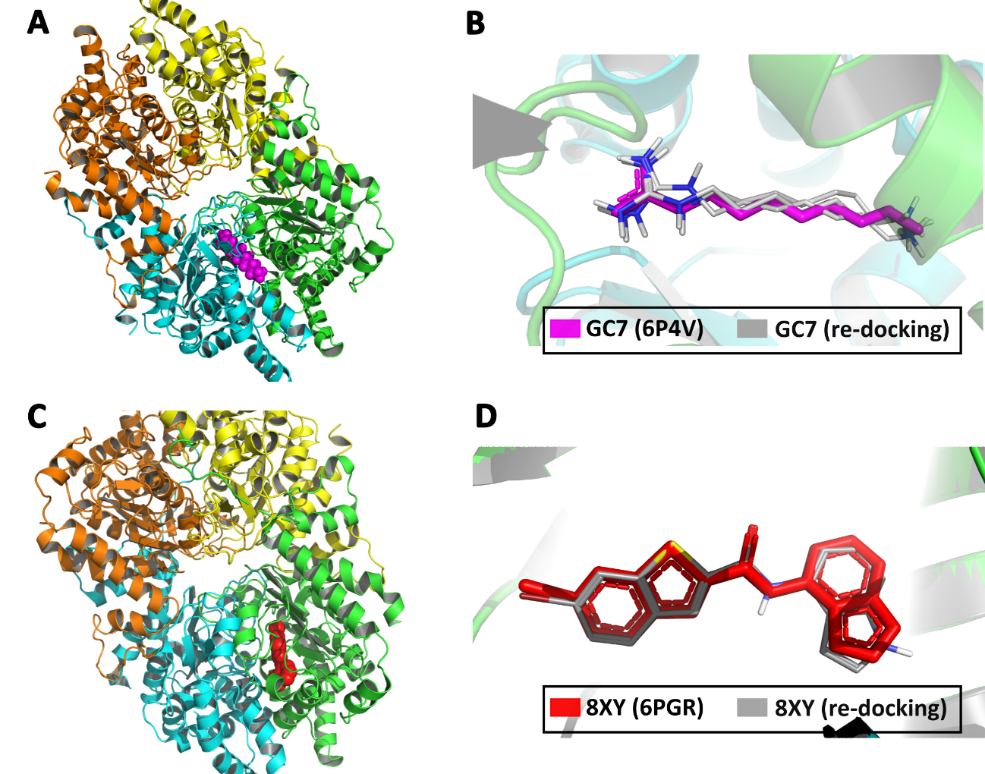
**

**S3 Fig.** Alignment of the re-docked ligands (gray) with its original co-

crystallized conformation.

Zoomed-out view of GC7 (pink) and 8XY (red) disposition in the tetramer

structures 6P4V (A) and PDB ID: 6PGR (C), respectively.

B and D show the overlay of the original ligands, GC7 (pink) and 8XY (red) with

the ligands after redocking (gray). Different colors represent individual chains in

the structure.
