# Supplementary material for: An experimental target-based platform in yeast for screening Plasmodium vivax deoxyhypusine synthase inhibitors
Source: PLoS Negl Trop Dis. 2024 Dec 2;18(12):e0012690. doi: 10.1371/journal.pntd.0012690 (PMC11637365; doi:10.1371/journal.pntd.0012690)
Supplement: S4 Fig — The binding mode and interaction profile of the orthosteric ligands N1 to N6 (A to F, respectively; in orange, labeled in the figure) and allosteric ligands (N7 to N9, G to H, respectively) in PvDHS residues (gray). The residues from PvDHS that interact with the compounds are identified by the three-letter amino acid code followed by number and chain. Different type of interactions is represented by different colors and lines, and their corresponding meaning are described at the bottom of the figure. (DOCX) [file pntd.0012690.s004.docx]

**
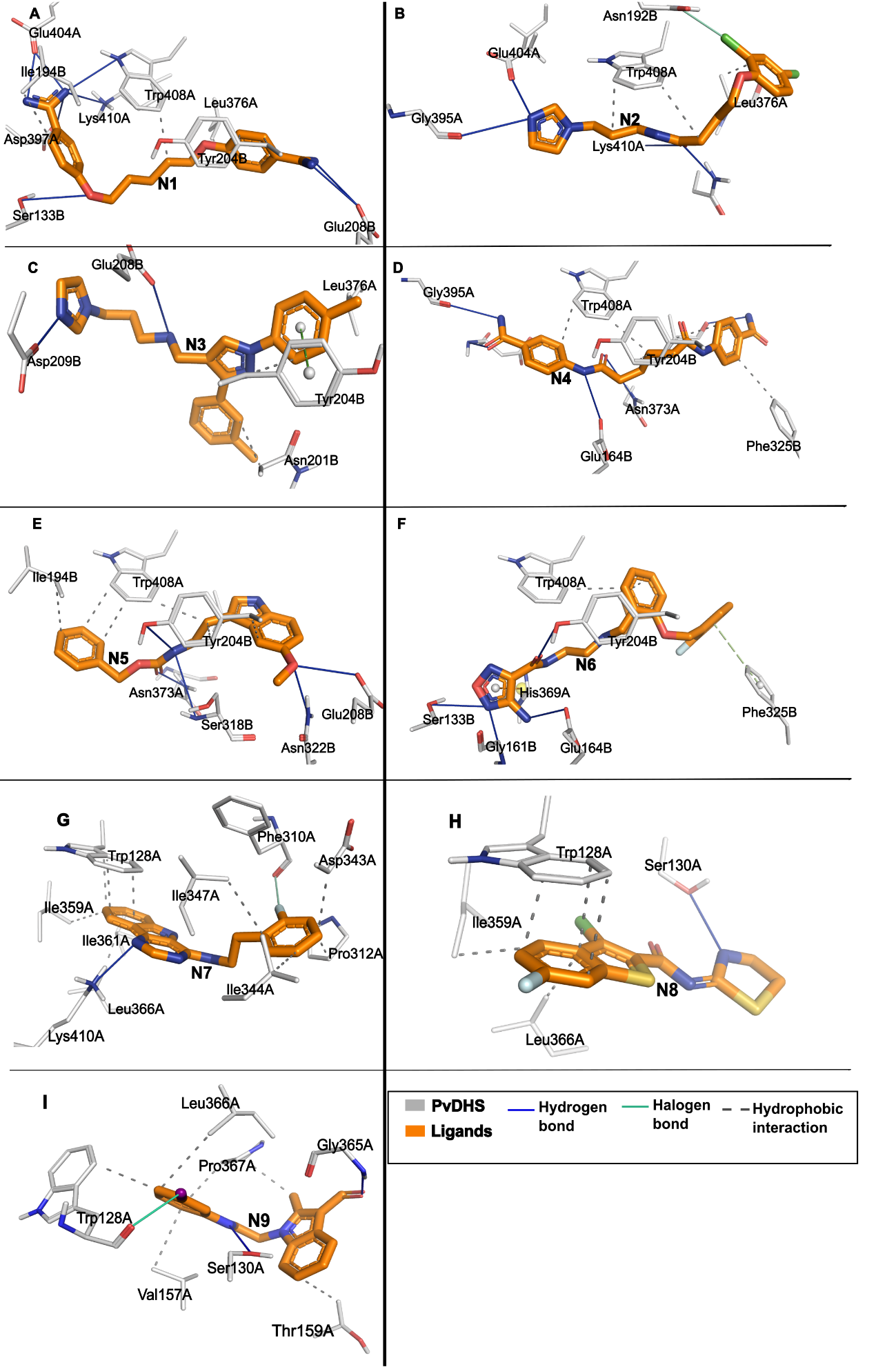
**

**S4 Fig.** Predicted binding mode of the selected compounds in PvDHS.

The binding mode and interaction profile of the orthosteric ligands N1 to N6 (A to F, respectively; in orange, labeled in the figure) and allosteric ligands (N7 to N9, G to H, respectively) in PvDHS residues (gray). The residues from PvDHS that interact with the compounds are identified by the three-letter amino acid code followed by number and chain. Different type of interactions is represented by different colors and lines, and their corresponding meaning are described at the bottom of the figure.
