# Supplementary material for: An experimental target-based platform in yeast for screening Plasmodium vivax deoxyhypusine synthase inhibitors
Source: PLoS Negl Trop Dis. 2024 Dec 2;18(12):e0012690. doi: 10.1371/journal.pntd.0012690 (PMC11637365; doi:10.1371/journal.pntd.0012690)
Supplement: S5 Fig — (A) Western blot detection of hypusinated eIF5A and total eIF5A from the S. cerevisiae strains wt, dys1Δ::HsDHS and dys1Δ::PvDHS (SFS01, SFS04 and SFS05, S2 Table). An antibody specific for S. cerevisiae DHS (ScDHS) was used to show the deletion of the endogenous gene in the strains replaced by HsDHS and PvDHS. (B) Inhibition of growth of P. vivax DHS-complemented by different methionine concentrations. (C) Western blot detection of hypusination levels after 10 h of methionine treatment (concentrations indicated in the figure). (D) Semi-quantitative graph reporting the percentage of hypusinated eIF5A (hypusinated eIF5A/total eIF5A *100) under the conditions indicated in the figure. Bars represent mean ± standard deviation (n = 3) of the percentage of hypusinated eIF5A. (DOCX) [file pntd.0012690.s005.docx]

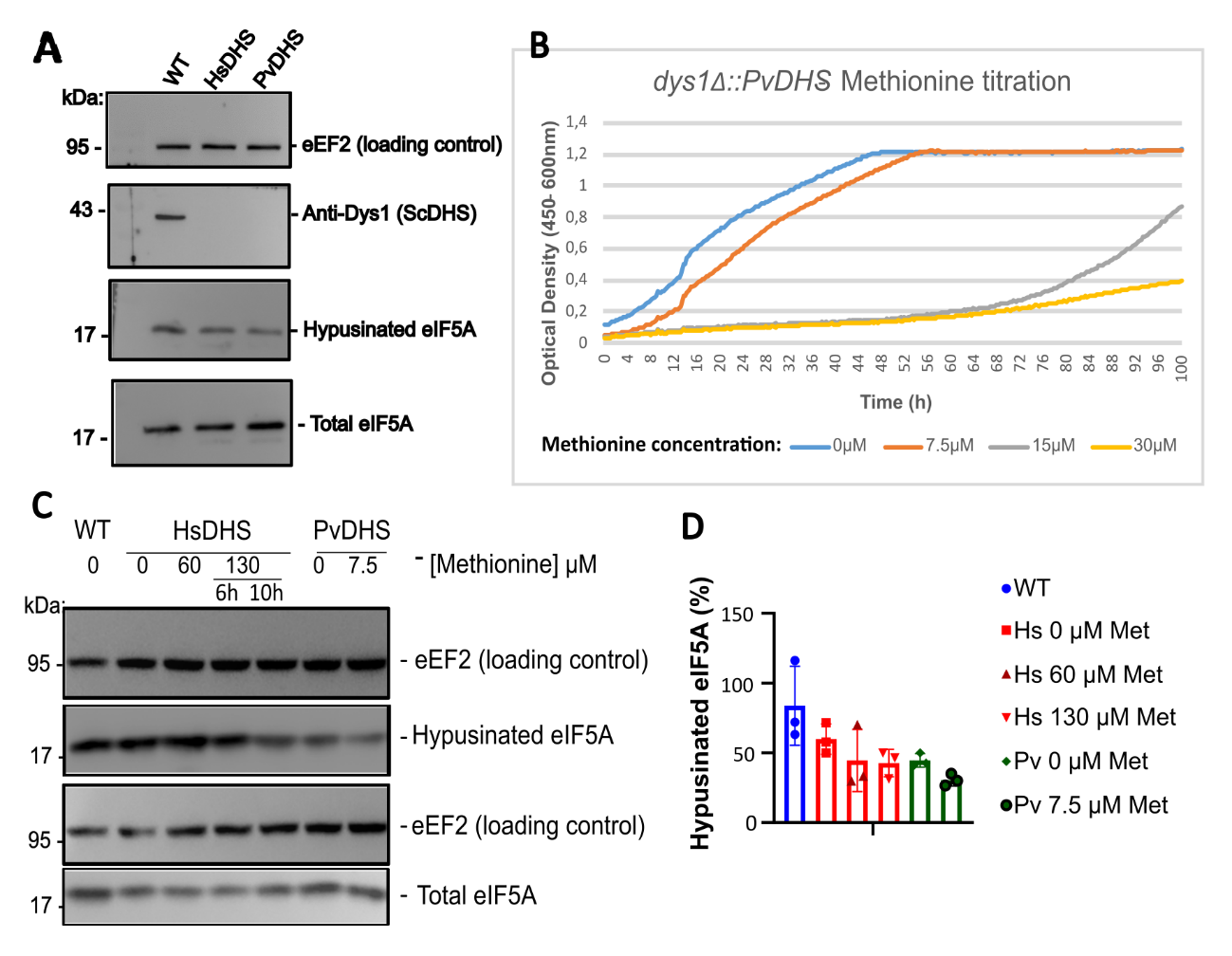


**S5 Fig.** Hypusination of yeast eIF5A by *H. sapiens* and *P. vivax* DHS-complemented strains.

(A) Western blot detection of hypusinated eIF5A and total eIF5A from the *S. cerevisiae* strains wt, *dys1Δ::HsDHS* and *dys1Δ::PvDHS* (SFS01, SFS04 and SFS05, Table S2). An antibody specific for *S. cerevisiae* DHS (ScDHS) was used to show the deletion of the endogenous gene in the strains replaced by HsDHS and PvDHS. (B) Inhibition of growth of *P. vivax* DHS-complemented by different methionine concentrations. (C) Western blot detection of hypusination levels after 10 h of methionine treatment (concentrations indicated in the figure). (D) Semi-quantitative graph reporting the percentage of hypusinated eIF5A (hypusinated eIF5A/total eIF5A *100) under the conditions indicated in the figure. Bars represent mean ± standard deviation (n = 3) of the percentage of hypusinated eIF5A.
