# Supplementary material for: An experimental target-based platform in yeast for screening Plasmodium vivax deoxyhypusine synthase inhibitors
Source: PLoS Negl Trop Dis. 2024 Dec 2;18(12):e0012690. doi: 10.1371/journal.pntd.0012690 (PMC11637365; doi:10.1371/journal.pntd.0012690)
Supplement: S6 Fig — The strain used was SFS05 (S2 Table). The growth measurements were carried out in the Eve robot (see Materials and methods) and it is given in arbitrary fluorescence units (AFU) (mean ± SD, n = 4). Cell cultures were grown in SC–met and 1.25% DMSO or 0, 25, 50, 100 and 200 μM concentrations of the respective compound tested (see legend). (DOCX) [file pntd.0012690.s006.docx]

**
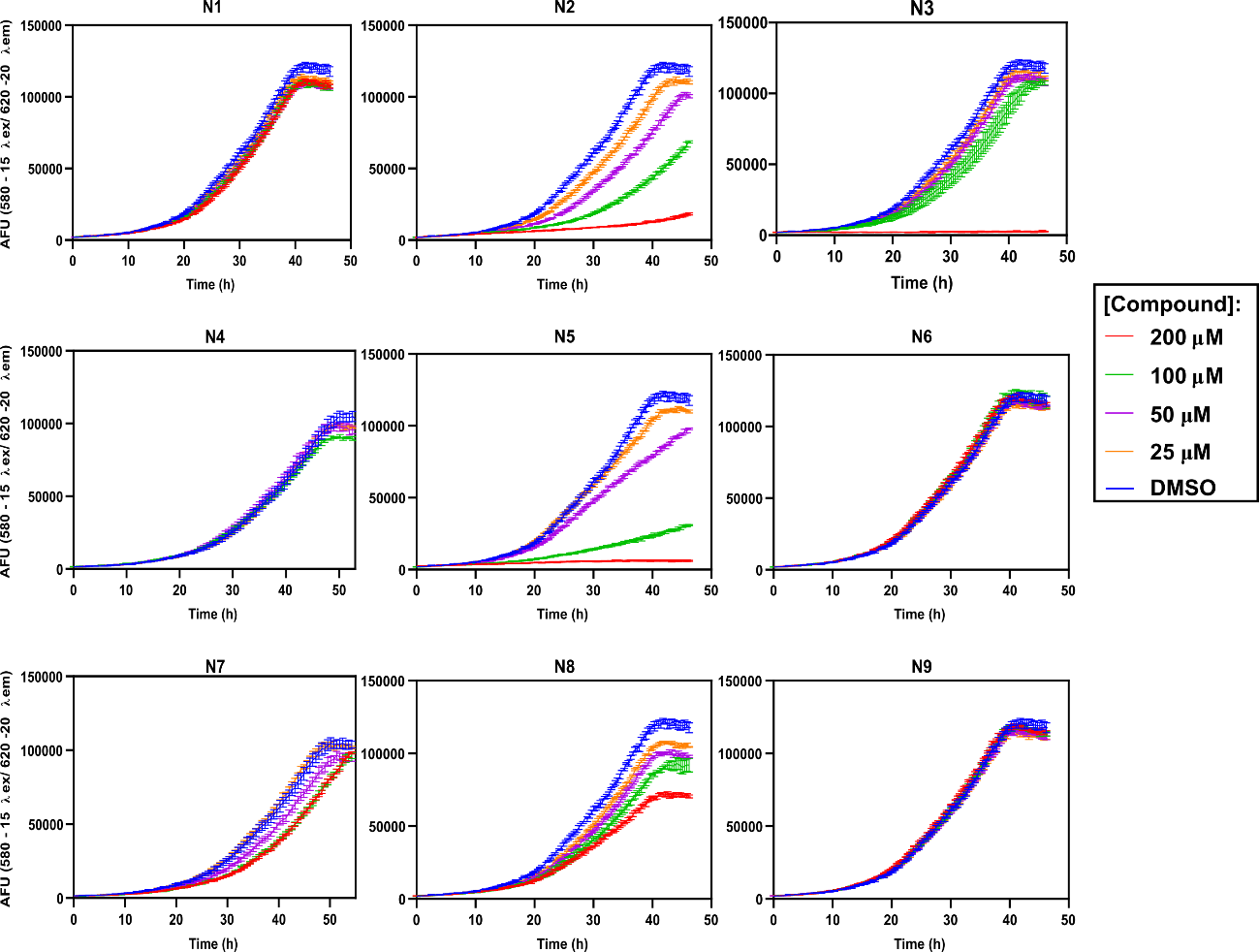
**

**S6 Fig.**  Growth of yeast DHS deleted strain complemented by PvDHS in the presence of compounds N1 to N9.

The strain used was SFS05 (S2 Table). The growth measurements were carried out in the Eve robot (see Materials and Methods) and it is given in arbitrary fluorescence units (AFU) (mean ± SD, n = 4). Cell cultures were grown in SC–met and 1.25 % DMSO or 0, 25, 50, 100 and 200 μM concentrations of the respective compound tested (see legend).
