# Supplementary material for: An experimental target-based platform in yeast for screening Plasmodium vivax deoxyhypusine synthase inhibitors
Source: PLoS Negl Trop Dis. 2024 Dec 2;18(12):e0012690. doi: 10.1371/journal.pntd.0012690 (PMC11637365; doi:10.1371/journal.pntd.0012690)
Supplement: S7 Fig — The strain used was SFS04 (S2 Table). The growth measurements, conducted in the Eve robot (see Materials and methods) are presented in arbitrary fluorescence units (AFU) and shown as mean ± SD, n = 4 replicates). Cell cultures were grown in SC with solvent alone (1.25% DMSO) or varying concentrations (0, 25, 50, 100 and 200 μM) of the respective tested compound, as indicated in the legend. (DOCX) [file pntd.0012690.s007.docx]

**
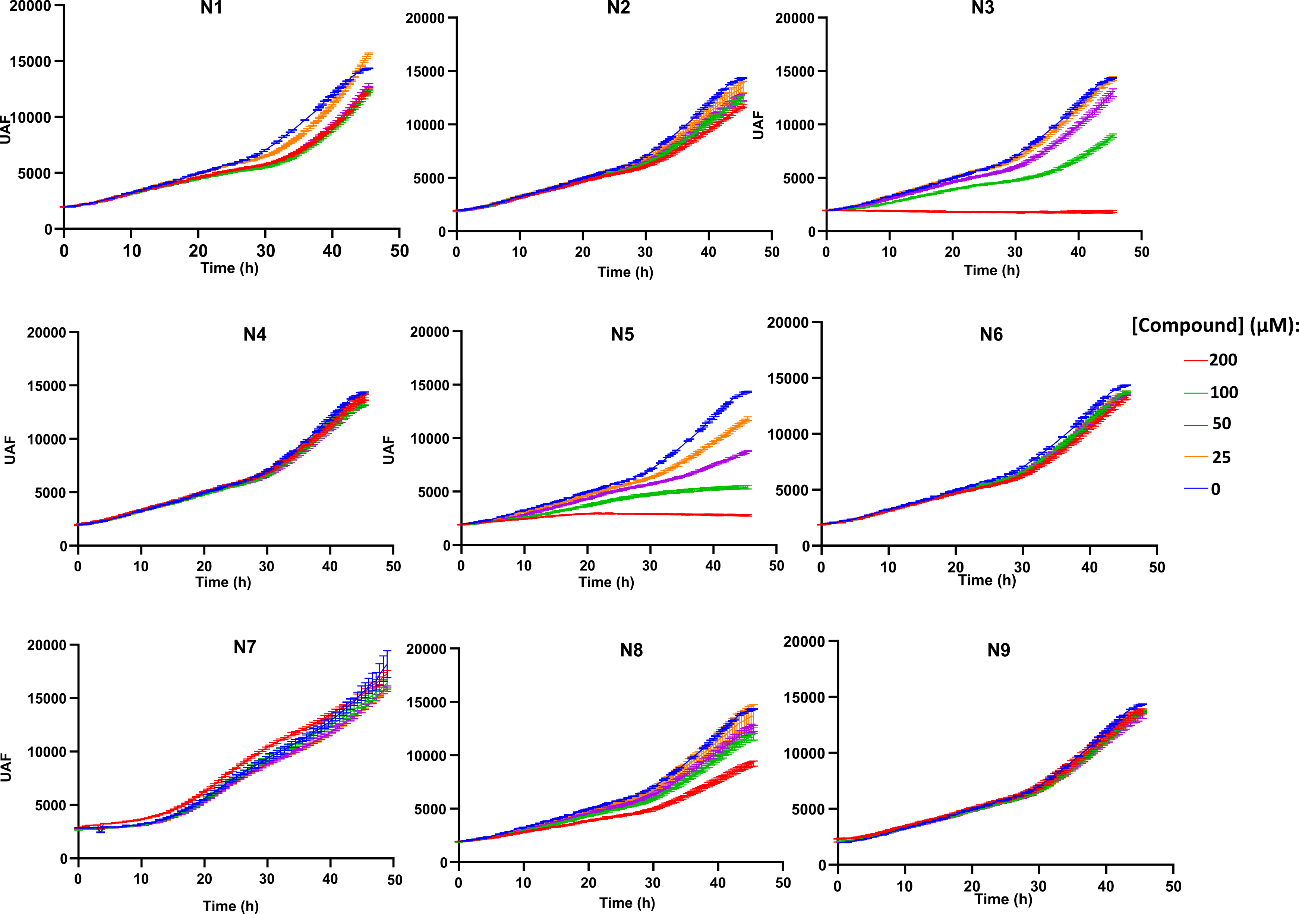
**

**S7 Fig.** Growth of yeast DHS deleted strain complemented by HsDHS in the presence of compounds N1 to N9.

The strain used was SFS04 (S2 Table). The growth measurements, conducted in the Eve robot (see Materials and Methods) are presented in arbitrary fluorescence units (AFU) and shown as mean ± SD, n = 4 replicates). Cell cultures were grown in SC with solvent alone (1.25 % DMSO) or varying concentrations (0, 25, 50, 100 and 200 μM) of the respective tested compound, as indicated in the legend.
