# Supplementary material for: An experimental target-based platform in yeast for screening Plasmodium vivax deoxyhypusine synthase inhibitors
Source: PLoS Negl Trop Dis. 2024 Dec 2;18(12):e0012690. doi: 10.1371/journal.pntd.0012690 (PMC11637365; doi:10.1371/journal.pntd.0012690)
Supplement: S8 Fig — The strain used was SFS01 (S2 Table). The growth measurements were carried out in the Eve robot (see Materials and methods) and it is given in arbitrary fluorescence units (AFU) (mean ± SD, n = 4). Cell cultures were grown in SC and solvent alone (1.25% DMSO) or varying concentrations or (25, 50, 100 and 200 μM) of the respective compound tested as indicated in the legend). (DOCX) [file pntd.0012690.s008.docx]

**
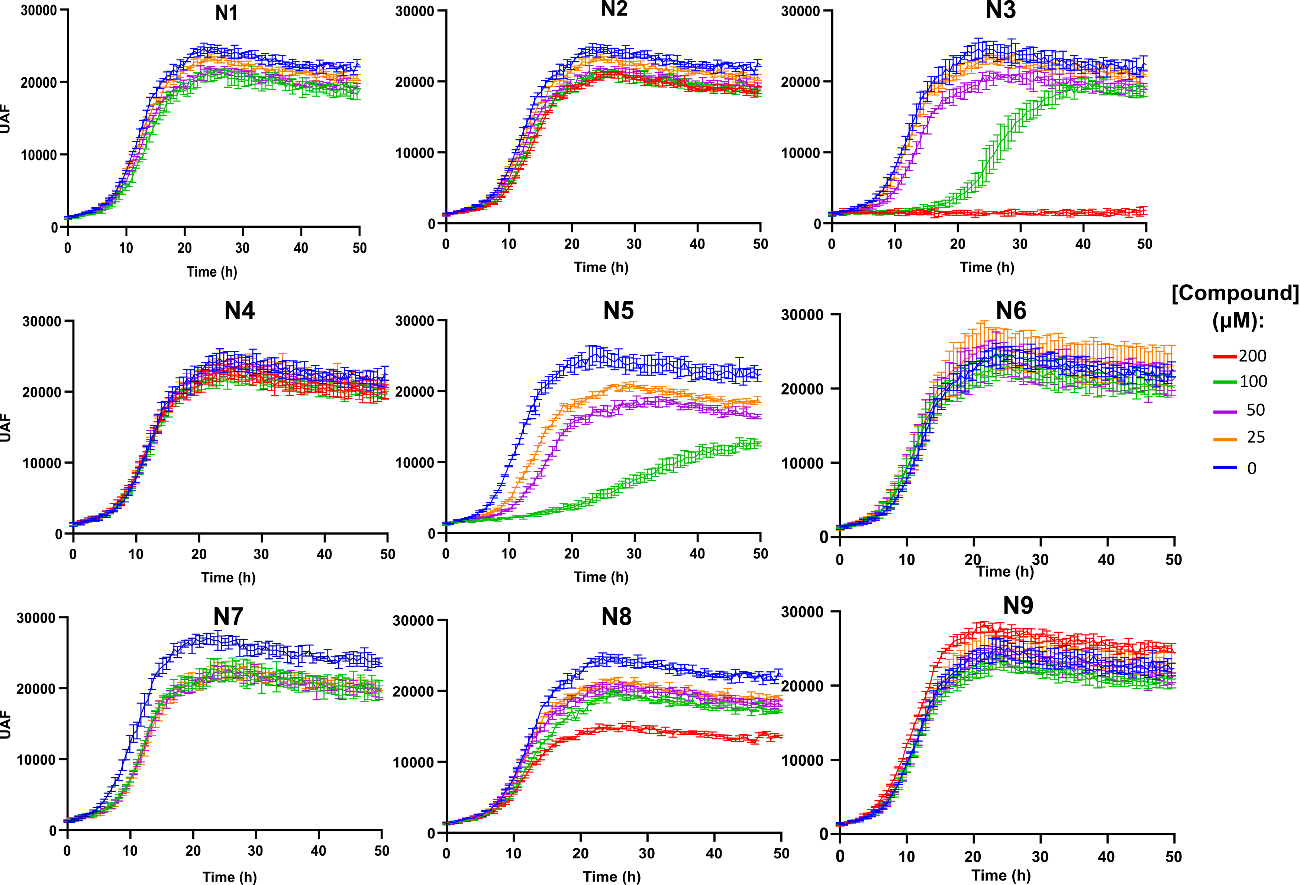
**

**S8 Fig.** Growth of yeast wt isogenic strain in the presence of compounds N1 to N9.

The strain used was SFS01 (S2 Table). The growth measurements were carried out in the Eve robot (see Materials and Methods) and it is given in arbitrary fluorescence units (AFU) (mean ± SD, n = 4). Cell cultures were grown in SC and solvent alone (1.25 % DMSO) or varying concentrations or (25, 50, 100 and 200 μM) of the respective compound tested as indicated in the legend).
