# Supplementary material for: An experimental target-based platform in yeast for screening Plasmodium vivax deoxyhypusine synthase inhibitors
Source: PLoS Negl Trop Dis. 2024 Dec 2;18(12):e0012690. doi: 10.1371/journal.pntd.0012690 (PMC11637365; doi:10.1371/journal.pntd.0012690)
Supplement: S9 Fig — Bars represent the relative growth score of S. cerevisiae wt or dys1Δ strains complemented by the PvDHS or HsDHS enzymes. The compound concentrations range from 25 to 200 μM. Statistical significance levels indicated as in Fig 2. (DOCX) [file pntd.0012690.s009.docx]

*
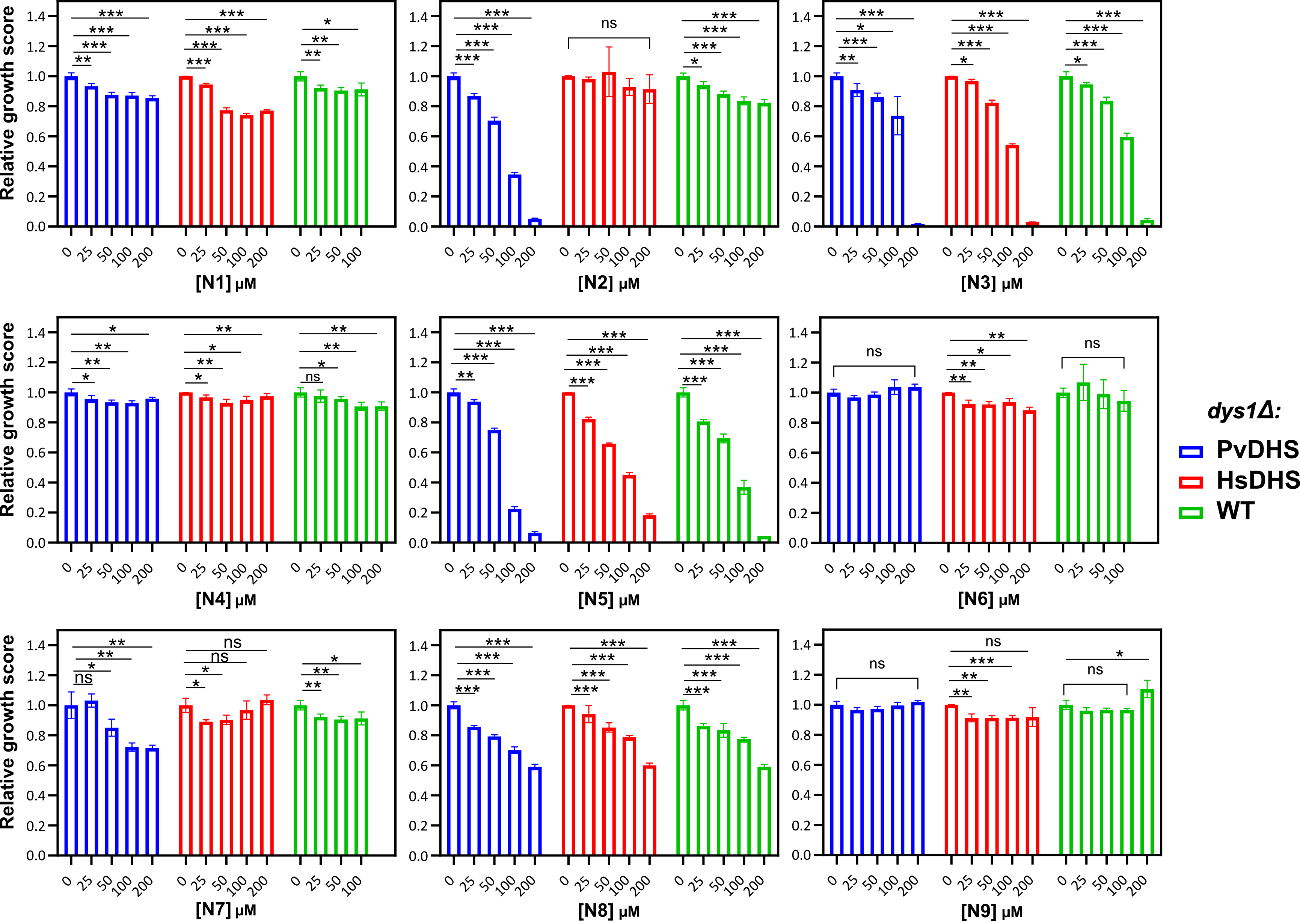
*

**S9 Fig.** Relative area under the curve extracted from growth curves.

Bars represent the relative growth score of S. cerevisiae wt or dys1Δ strains complemented by the PvDHS or HsDHS enzymes. The compound concentrations range from 25 to 200 µM. Statistical significance levels indicated as in Fig. 2.
