# Supplementary material for: An experimental target-based platform in yeast for screening Plasmodium vivax deoxyhypusine synthase inhibitors
Source: PLoS Negl Trop Dis. 2024 Dec 2;18(12):e0012690. doi: 10.1371/journal.pntd.0012690 (PMC11637365; doi:10.1371/journal.pntd.0012690)
Supplement: S10 Fig — The strain used was SFS05 (S2 Table). The growth measurements were carried out in the Eve robot (see Materials and methods) and it is given in arbitrary fluorescence units (AFU) (mean ± SD, n = 4). Cell cultures were grown in SC–met and 1.25% DMSO or 25 μM of the respective compound tested (see legend). The compounds are named according to the Pathogen box compound ID. (DOCX) [file pntd.0012690.s010.docx]

**
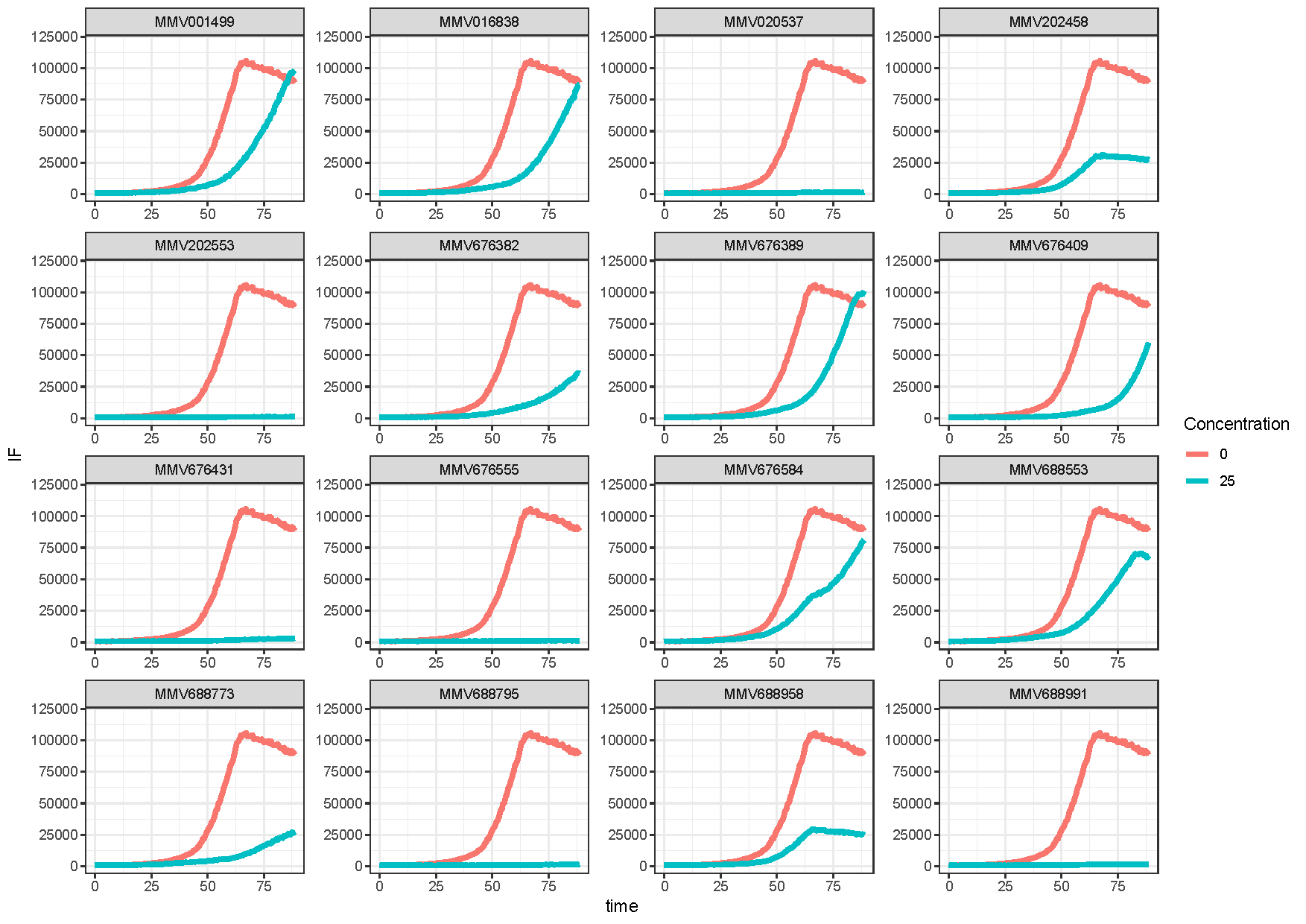
**

**S10 Fig.** Growth reduction of PvDHS-complemented strain caused by compounds from the Pathogen Box.

The strain used was SFS05 (S2 Table). The growth measurements were carried out in the Eve robot (see Materials and Methods) and it is given in arbitrary fluorescence units (AFU) (mean ± SD, n = 4). Cell cultures were grown in SC–met and 1.25 % DMSO or 25 μM of the respective compound tested (see legend). The compounds are named according to the Pathogen box compound ID.
