# Supplementary material for: An experimental target-based platform in yeast for screening Plasmodium vivax deoxyhypusine synthase inhibitors
Source: PLoS Negl Trop Dis. 2024 Dec 2;18(12):e0012690. doi: 10.1371/journal.pntd.0012690 (PMC11637365; doi:10.1371/journal.pntd.0012690)
Supplement: S11 Fig — The inhibitory potential of different compounds was tested at concentrations ranging from 0.01 to 80 μM and the inhibition of parasitemia was measured after 72 hours of incubation. The growth inhibition values were expressed as percentages relative to the drug-free control and EC50 value were calculated by plotting log10 of compound concentrations vs growth inhibition (expressed as percentage relative to the drug-free control). The experiments were carried out in three independent assays. (DOCX) [file pntd.0012690.s011.docx]

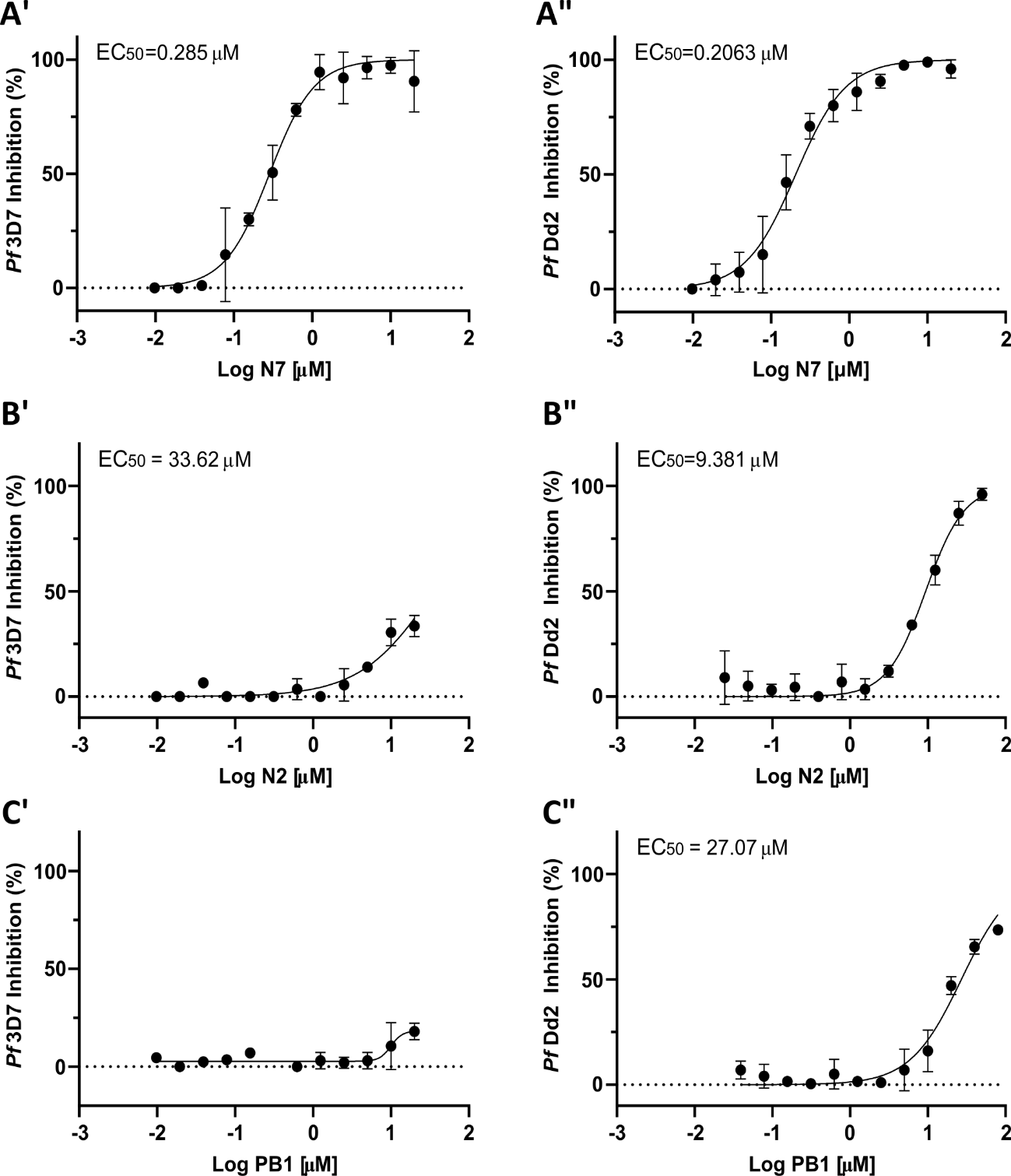


**S11 Fig. *In vitro* growth inhibition of asexual blood stage *P. falciparum* (3D7 or Dd2) for compounds N7, N2 and PB1.** The inhibitory potential of different compounds was tested at concentrations ranging from 0.01 to 80 μM and the inhibition of parasitemia was measured after 72 hours of incubation. The growth inhibition values were expressed as percentages relative to the drug-free control and EC_50_ value were calculated by plotting log_10_ of compound concentrations vs growth inhibition (expressed as percentage relative to the drug-free control). The experiments were carried out in three independent assays.
