# Supplementary material for: An experimental target-based platform in yeast for screening Plasmodium vivax deoxyhypusine synthase inhibitors
Source: PLoS Negl Trop Dis. 2024 Dec 2;18(12):e0012690. doi: 10.1371/journal.pntd.0012690 (PMC11637365; doi:10.1371/journal.pntd.0012690)
Supplement: S12 Fig — The compounds were tested at concentrations ranging from 0.1 to 100 μM, for 24 h in MCF7 and HepG2 cells, as indicated in the panels). Cell viability (%) was calculated as the ratio between cell incubated with compound and cell incubated with DMSO. Each point represents the mean ± standard deviation, with n = 2 replicates. (DOCX) [file pntd.0012690.s012.docx]

**
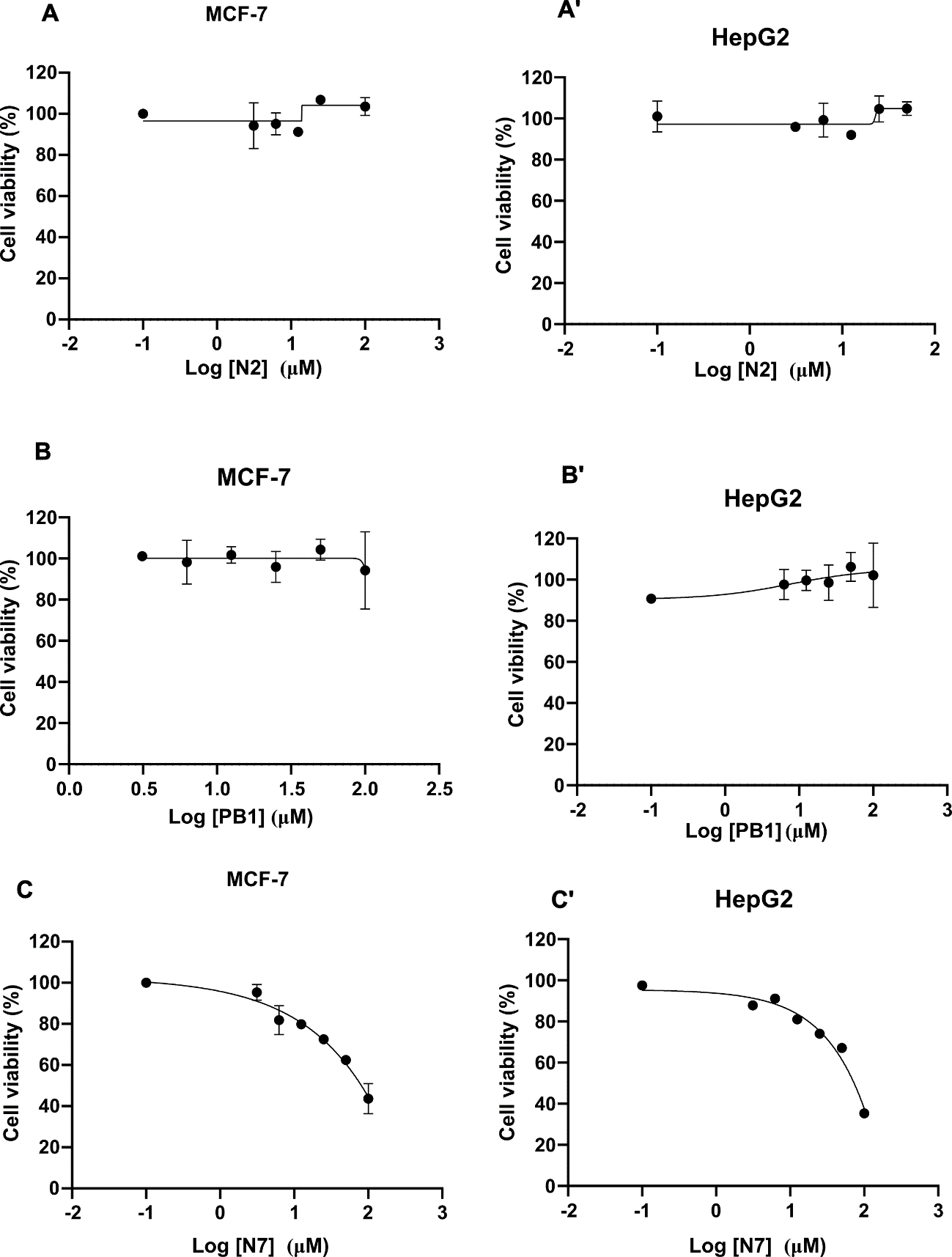
**

**S12 Fig**. Cell viability analysis after treatment with compounds N2, N7

and PB1.

The compounds were tested at concentrations ranging from 0.1 to 100 µM, for

24 h in MCF7 and HepG2 cells, as indicated in the panels). Cell viability (%)

was calculated as the ratio between cell incubated with compound and cell

incubated with DMSO. Each point represents the mean ± standard deviation,

with n = 2 replicates.
