# Supplementary material for: An experimental target-based platform in yeast for screening Plasmodium vivax deoxyhypusine synthase inhibitors
Source: PLoS Negl Trop Dis. 2024 Dec 2;18(12):e0012690. doi: 10.1371/journal.pntd.0012690 (PMC11637365; doi:10.1371/journal.pntd.0012690)
Supplement: S1 Table — (DOCX) [file pntd.0012690.s013.docx]

**S1 Table.** Plasmids used in this study.

| **Plasmid** | **Features** | **Derived from** | **Source** |
| --- | --- | --- | --- |
| pWS082 | sgRNA entry vector; *Amp^R^* | - | Tom Ellis (Addgene plasmid # 90516) |
| pWS082-CAN1 sgRNA | CAN1 sgRNA | pWS082 | This work |
| pWS082-dys1 sgRNA | DYS1 sgRNA | pWS082 | This work |
| pWS158 | Cas9 gap repair vector; *URA3; Kan^R^* | - | Tom Ellis (Addgene plasmid # 90517) |
| pWS172 | Cas9 gap repair vector; *HIS3; Kan^R^* | - | Tom Ellis (Addgene plasmid # 90519) |
| pCM188-MET3 | *CEN; URA3; Amp^R^; MET3pr* (methionine repressible) | - | [1] |
| pCM188-MET3-HsDHS | *Homo sapiens* DHS | pCM188-MET3 | This work |
| pCM188-MET3-PvDHS | *Plasmodium vivax* DHS | pCM188-MET3 | This work |
| yEp_CHERRY_HIS3 | *mCherry; HIS3* | - | [2] |
| yEp_Sapphire_HIS3 | *Sapphire; HIS3* | - | [2] |

Reference

1. Alalam H, Sigurdardóttir S, Bourgard C, Tiukova IA, King RD, Grøtli M, Sunnerhagen P. A genetic trap in yeast for inhibitors of the SARS-CoV-2 main protease. mSystems. 2021; 6:e01087-21

2. Bilsland E, Sparkes A, Williams K, Moss HJ, de Clare M, Pir P, et al. Yeast-based automated high-throughput screens to identify anti-parasitic lead compounds. Open Biol. 2013; 3:120158.
