# Supplementary material for: An experimental target-based platform in yeast for screening Plasmodium vivax deoxyhypusine synthase inhibitors
Source: PLoS Negl Trop Dis. 2024 Dec 2;18(12):e0012690. doi: 10.1371/journal.pntd.0012690 (PMC11637365; doi:10.1371/journal.pntd.0012690)
Supplement: S2 Table — (DOCX) [file pntd.0012690.s014.docx]

**S2 Table.** *Saccharomyces cerevisiae* strains used in this study.

| Strain | Genotype | Source |
| --- | --- | --- |
| HA_SC_1352control | *MATα his3Δ1 leu2Δ0 met15Δ0 ura3Δ0 pdr1Δ::NatMX pdr3Δ::MET17 snq2Δ::KI.LEU2* | [1] |
| SFS01 | HA_SC_1352control - *can1::mCherry* | This work |
| SFS02 | HA_SC_1352control - *can1::Sapphire* | This work |
| SFS04 | SFS02 - *dys1Δ::MET3pr-HsDHS-CYCt* | This work |
| SFS05 | SFS01 - *dys1Δ::MET3pr-PvDHS-CYCt* | This work |

Reference

1. Alalam H, Sigurdardóttir S, Bourgard C, Tiukova IA, King RD, Grøtli M, Sunnerhagen P. A genetic trap in yeast for inhibitors of the SARS-CoV-2 main protease. mSystems. 2021; 6:e01087-21
