# Supplementary material for: An experimental target-based platform in yeast for screening Plasmodium vivax deoxyhypusine synthase inhibitors
Source: PLoS Negl Trop Dis. 2024 Dec 2;18(12):e0012690. doi: 10.1371/journal.pntd.0012690 (PMC11637365; doi:10.1371/journal.pntd.0012690)
Supplement: S3 Table — (DOCX) [file pntd.0012690.s015.docx]

**S3 Table.** Oligonucleotides used in this study.

| Primer | Sequence 5’-3’ | Comment |
| --- | --- | --- |
| HsDHS F | GGCGGATCCATGCATCATCACCATCACCACATGGAAGGTTCTTTGGAAAGGGAAG | Amplification of HsDHS + 6xHis + restriction site |
| HsDHS R | CGCTGCAGTCAGTCCTCGTTCTTTTCGTGC | Amplification of HsDHS |
| PvDHS F | CGCGGATCCATGCATCATCACCATCACCACATGACTAATCAAGGTGCCTTCAAAGAAGTTAACA | Amplification of PvDHS + 6xHis + restriction site |
| PvDHS R | GACTGCAGTCACCTCAATTGTGCTTCACCAG | Amplification of PvDHS |
| pCM F | CGTAGGTGAACTGATTGAGAAAAAT | DNA sequencing |
| CYC1t R | GGCGTGAATGTAAGCGTGAC | DNA sequencing |
| DYS1_A | GGAATGTACACATTTCAAATTAGGG | Deletion check *DYS1* |
| DYS1_D | CGTAGGTGAACTGATTGAGAAAAAT | Deletion check *DYS1* |
| PDR5 F | GCCTCTTTGTTGTTTACAATGTCTT | Deletion check *PDR5* |
| PDR5 R | TCACACTAAATGCTGATGCCTATAA | Deletion check *PDR5* |
| sgRNA CAN1 F | GACTTTGATACGTTCTCTATGGAGGA | Guide RNA *locus* *CAN1* |
| sgRNA CAN1 R | AAACTCCTCCATAGAGAACGTATCAA | Guide RNA *locus* *CAN1* |
| sgRNA DYS1 F | GACTTTACGAATCCTCCGTATTGTAC | Guide RNA *locus* *DYS1* |
| sgRNA DYS1 R | AAACGTACAATACGGAGGATTCGT | Guide RNA *locus* *DYS1* |
| dys1-tADH1 Repair 3 F | GCTTAGTTAACTTATATACAAGTCAAAGCAAAAAAGATGTCCGATATCAACGcctaggaattggagcgacctcatg | Integration of mCherry/Sapphire into *CAN1 locus* |
| dys1-MET3p Repair 3 F | GCTTAGTTAACTTATATACAAGTCAAAGCAAAAAAGATGTCCGATATCAACGctttagtactaacagagacttttgtcac | Integration of HsDHS/PvDHS under control of MET3pr into *DYS1 locus* |
| dys1-tCYC Repair 3 R | CTCTCATCAATTCTTAACTTTTTTGATTGGTTTACCACTGGCAAAGGTAGCAGCAACcttcgagcgtcccaaaaccttctc | Integration of HsDHS/PvDHS under control of MET3pr into *DYS1 locus* |
| Dys1 F | TCGTGTTTTGCTTAGTTAACTTATATACAA | DNA sequencing/ *DYS1* deletion check |
