# Supplementary material for: An experimental target-based platform in yeast for screening Plasmodium vivax deoxyhypusine synthase inhibitors
Source: PLoS Negl Trop Dis. 2024 Dec 2;18(12):e0012690. doi: 10.1371/journal.pntd.0012690 (PMC11637365; doi:10.1371/journal.pntd.0012690)
Supplement: S4 Table — (DOCX) [file pntd.0012690.s016.docx]

**S4 Table. Synthetic compounds used in this study.**

| **2D Structures** | **Generic name** | **Compound ID** | **Compound name** | **Supplier** |
| --- | --- | --- | --- | --- |
|  | GC7 (DHS competitive inhibitor) | GC7 | N1-Guanyl-1,7-Diaminoheptane | Merck |
| 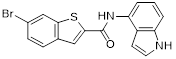 | 8XY (DHS allosteric inhibitor) | 8XY/11g | 6-bromo-N-(1H-indol-4-yl)-1-benzothiophene-2-carboxamide | Compound used only for molecular docking |
|  | N1 | GNF-Pf-3680 | 4-[5-(4-carbamimidoylphenoxy)pentoxy]benzenecarboximidamide | Apexbio Technology LLC |
|  | N2 | GNF-Pf-744 | 4-(2,4-dichlorophenoxy)-N-(3-imidazol-1-ylpropyl)butanamide | ChemBridge Corporation |
|  | N3 | TCMDC-124630 | 3-imidazol-1-yl-N-[[3-(3-methylphenyl)-1-(4-methylphenyl)pyrazol-4-yl]methyl]propan-1-amine | ChemBridge Corporation |
|  | N4 | CHEMBL1081521 | N,N'-bis(4-carbamoylphenyl)hexanediamide | ChemBridge Corporation |
|  | N5 | ZINC518875 | benzyl N-[2-(5-methoxy-1H-indol-3-yl)ethyl]carbamate | InterBioScreen Ltd. |
|  | N6 | CHEMBL1305755 | 4-amino-N-[2-[[2-[(2-fluorophenyl)methoxy]phenyl]methylamino]ethyl]-1,2,5-oxadiazole-3-carboxamide;hydrochloride | Vitas-M Laboratory, Ltd. |
|  | N7 | GNF-Pf-3738 | N-[2-(2-fluorophenyl)ethyl]-5H-pyrimido[5,4-b]indol-4-amine | InterBioScreen Ltd. |
|  | N8 | GNF-Pf-4241 | 2-(3-formyl-2-methylindol-1-yl)-N-(3-iodophenyl)acetamide | ChemBridge Corporation |
|  | N9 | CHEMBL3493253 | 1-[2-(furan-2-yl)-2-pyrrolidin-1-ylethyl]-3-(2-imidazol-1-ylethyl)urea | ENAMINE Ltd. |
|  | PB1 | MMV676584 | 3-chloro-N-(4,5-dihydro-1,3-thiazol-2-yl)-6-fluoro-1-benzothiophene-2-carboxamide | Pathogen Box / New batch from ChemBridge Corporation |
|  | PB2 | MMV688553 | 4-(1,3-benzodioxol-5-ylmethyl)-N-(furan-3-ylmethyl)piperazine-1-carboxamide | Pathogen Box / New batch from ChemBridge Corporation |
