# Supplementary material for: An experimental target-based platform in yeast for screening Plasmodium vivax deoxyhypusine synthase inhibitors
Source: PLoS Negl Trop Dis. 2024 Dec 2;18(12):e0012690. doi: 10.1371/journal.pntd.0012690 (PMC11637365; doi:10.1371/journal.pntd.0012690)
Supplement: S1 Text — (DOCX) [file pntd.0012690.s017.docx]

**S1 Text. Synthetic DHS genes with codon usage optimized for expression in *Saccharomyces cerevisiae.***

The coding region from each gene was flanked by *Bam H*I and *Pst* I restriction sites (highlighted in yellow).

**HsDHS**

GGATCCATGGAAGGTTCTTTGGAAAGGGAAGCTCCAGCTGGTGCATTGGCTGCTGTTTTGAAACATTCTTCTACTTTGCCACCAGAATCTACCCAAGTTAGAGGTTACGATTTTAACAGAGGTGTTAACTACAGAGCTTTGTTGGAAGCTTTTGGTACTACTGGTTTCCAAGCTACTAATTTCGGTAGAGCTGTTCAACAAGTTAACGCCATGATTGAAAAGAAGTTGGAACCATTGTCTCAAGACGAAGATCAACATGCTGATTTGACTCAATCTAGAAGGCCATTGACATCTTGCACTATTTTCTTGGGTTACACCTCCAACTTGATCTCTTCTGGTATTAGAGAAACCATCAGATACTTGGTCCAACACAACATGGTTGATGTTTTGGTTACTACAGCTGGTGGTGTTGAAGAGGATTTGATTAAGTGTTTGGCTCCAACTTACTTGGGTGAATTTTCCTTGAGAGGCAAAGAATTGAGAGAGAACGGTATTAACAGAATCGGCAATTTGTTGGTCCCAAACGAAAATTACTGCAAGTTCGAAGATTGGTTGATGCCAATCTTGGATCAAATGGTCATGGAACAAAACACCGAAGGTGTTAAGTGGACTCCATCTAAAATGATTGCCAGATTGGGCAAAGAAATCAACAATCCAGAATCCGTTTATTACTGGGCCCAAAAGAATCATATCCCAGTTTTTTCACCAGCTTTGACCGATGGTTCTTTAGGTGATATGATCTTCTTCCACTCCTACAAAAATCCAGGTTTGGTTTTGGATATCGTCGAGGACTTGAGATTGATTAACACCCAAGCTATTTTCGCTAAGTGCACCGGTATGATTATCTTAGGTGGTGGTGTAGTCAAACATCATATTGCTAATGCTAACTTGATGAGAAACGGTGCTGATTACGCTGTTTACATTAACACTGCTCAAGAATTCGACGGTTCTGATTCAGGTGCTAGACCAGATGAAGCTGTTTCTTGGGGTAAAATTAGAGTTGATGCTCAACCAGTTAAGGTTTACGCTGATGCTTCTTTGGTTTTCCCATTATTGGTTGCTGAAACCTTCGCTCAAAAGATGGATGCTTTTATGCACGAAAAGAACGAGGACTGATGACTGCAGGGTACCTGGAG

**PvDHS**

GGATCCATGACTAATCAAGGTGCCTTCAAAGAAGTTAACAAGATCAGGTCTGAATCCGATGATGGTGAATCTTCTGACGAAAAGTCTGGTATTGAAGATGCCAAGTCATCCGTTTTCGTTAAGTCCAACAAAATCCCAGAAAACACCGATGTTGTTAAGGGTATCAACTTCGAAGAAGAAGTCAACTTGCACCAATTCGTTAACCAGTATAAGTACATGGGTTTCCAAGCTACCAACTTAGGTATTGGTATCGATGAGGTCAACAAGATGATCCATTTTAAGTATGCTGAAGGTGGTGAAGGTACTCAAGATGGTCATGATAATGATCACGATCAAGATTCCGATGACGAAAGACAAGCTTTGCCAAAGAAAAAGAAGTGCTTGATCTGGTTGTCTTTCACCTCCAATATGATCTCTTCTGGTTTGAGAGAAATCTTCGTCTACCTGGCTAAGAAAAAGTTCATCGATGTTGTCGTTACTACTGCTGGTGGTGTTGAAGAGGATATTATCAAGTGTTTCTCCAAGACTTACTTGGGCGATTTTAACTTGAACGGTAAGAAGTTGAGAAAGAAAGGTTGGAACAGAATCGGCAATTTGATTGTCCCAAACGATAACTACTGCAAGTTCGAAGATTGGTTGCAGCCTTTGTTGAACAAGATGTTGCATGAACAGAACCGTAAGAACGAAGAGTTGTTCTTGAGAAAGTTGGACAAAAGACGTAGAGGTGGTGGTCATGGTGGTGAAAGGGAACCACCATCTCCACCTCCACATACACCACATGCTCCATCACCACCAAGTCCATGTGATTCTTCAGATGAAGATGAATCCGACATGTTCTACTTGTCTCCATCTGAATTCATCGACAAATTGGGCGAAGAAATCAACGACGAATCTTCTTTGATATACTGGTGCCACAAGAACGATATTCCAGTTTTTTGTCCAGGTTTGACCGATGGTTCTTTGGGTGATAATTTGTTCTTCCACAATTACGGCAAGAAGATCAAAAACAACCTGATCCTGGATATCGTCAAGGACATCAAGAAGATTAACTCATTGGCTCTGAACTGCAAGAAGTCCGGTATTATCATTTTAGGTGGTGGCTTGCCAAAACATCATGTCTGTAATGCTAACTTGATGAGAAACGGTGCTGATTTCGCTGTTTACGTTAATACTGCTAACGAATACGACGGTTCTGATTCTGGTGCTAACACTACTGAAGCTTTGTCTTGGGGTAAAATCAAAGCTGGTCATACCAACAACCACGTTAAGGTTTTTGGTGATGCTACCATTTTGTTCCCATTGATGGTTTTGAACACCTTCTACTTGCATGACAGAGGTGGTAGACATAATTCTGGTGAAGCACAATTGAGGTGACTGGAG
