# Supplementary material for: An experimental target-based platform in yeast for screening Plasmodium vivax deoxyhypusine synthase inhibitors
Source: PLoS Negl Trop Dis. 2024 Dec 2;18(12):e0012690. doi: 10.1371/journal.pntd.0012690 (PMC11637365; doi:10.1371/journal.pntd.0012690)
Supplement: Attachment — Submitted filename: Rebuttal.pdf [file pntd.0012690.s018.pdf]

Göteborg, June 10, 2024

Response from editor Charles L. Jaffe, PLoS Neglected Tropical Diseases on Nov 29, 2023:

Dear Prof. Sunnerhagen,

Thank you very much for submitting your manuscript "An experimental target-based platform in yeast for screening Plasmodium vivax deoxyhypusine synthase inhibitors" (PNTD-D-23-01497) for consideration at PLOS Neglected Tropical Diseases. Based on our initial assessment, we regret that we will not be pursuing this manuscript for publication at PLOS Neglected Tropical Diseases.

While interesting, the editorial board felt that this paper is not suitable for publication in PLoS NTD in its present form. If the authors include additional experiments showing that the DHS inhibitors, identified using the yeast platform, also inhibit Plasmodium vivax growth in vitro we would be happy to consider your paper for publication.

We are sorry that we cannot be more positive on this occasion. We very much appreciate your wish to present your work in one of PLOS's Open Access publications. Thank you for your support, and we hope that you will consider PLOS Neglected Tropical Diseases for other submissions in the future.

Charles L. Jaffe, Ph.D.
Section Editor
PLOS Neglected Tropical Diseases

Rebuttal

General comments

Thank you for responding to our submission. It was not entirely obvious to me how to interpret this letter. You asked us to provide data on inhibition of *Plasmodium vivax in vitro* for the identified compounds.

Taken literally, this is not feasible as there is no continuous *in vitro* culture system established for *P. vivax*. This is in contrast to *P. falciparum*, where the asexual erythrocyte stage can be cultured in the lab indefinitely.

The only recourse for *P. vivax* is culture *ex vivo* with blood samples taken directly from infected humans. Since *P. vivax* is only viable *ex vivo* for a few days (~48 h), patient-sourced fresh parasites have to be obtained frequently. This means in practice that any experiments with *P. vivax* can only be conducted in a region endemic for this species, and in the framework of an established collaboration with a local clinic.

The experimental data on the effect of a compound on a human malaria parasite that can be obtained in a laboratory environment distant from regions where *P. vivax* infections are prevalent, are from *P. falciparum*. *In vitro* sensitivity assays on *P. falciparum* asexual blood stage are by far the most common way early in the drug discovery process to demonstrate antiplasmodial activity of a compound

targeting a *P. vivax* protein, as most molecules active against one of these *Plasmodium* species are also active against the other.

Changes in the new version

We have now performed growth inhibition assays *in vitro* with the new compounds on the asexual erythrocyte stage on two established laboratory lines of *P. falciparum*; 3D7 (chloroquine sensitive) and Dd2 (chloroquine resistant). Our results show that the three molecules display EC_{50} values against both lines between ~200 nM and ~30 μM.

These new data are shown in Results, text in the next to the last section, and in Table 2. Dose-response curves are shown in Table S11. The data is also mentioned in the Discussion.

Cytotoxicity data from two human cell lines (MCF7 and HepG2) was already present in the previous version. Two of the compounds show no detectable toxicity at the concentrations tested, while the third displayed moderate toxicity (IC_{50} ~30 μM).

Other than that, there are no changes in the new version beside some minor trimming and compaction of sentences throughout the manuscript.

We hope that with this new data, you will consider our manuscript for publication in PLoS Neglected Tropical Diseases.

Sincerely,

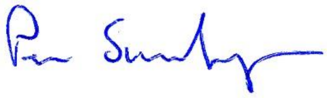

Per Sunnerhagen, Professor in Molecular Biology
Department of Chemistry and Molecular Biology, University of Gothenburg
S-405 30 Göteborg, Sweden
Phone: +46-31-786 3830
E-mail: Per.Sunnerhagen@cmb.gu.se
