# Supplementary material for: An experimental target-based platform in yeast for screening Plasmodium vivax deoxyhypusine synthase inhibitors
Source: PLoS Negl Trop Dis. 2024 Dec 2;18(12):e0012690. doi: 10.1371/journal.pntd.0012690 (PMC11637365; doi:10.1371/journal.pntd.0012690)
Supplement: Attachment — Submitted filename: Rebuttal.docx [file pntd.0012690.s019.docx]

**Replies to reviewers’ comments**

**Methods**

*Reviewer #1:*

*1. The main concern on this manuscript is the incomplete characterisation of the system.*

*As the pair of yeast strains expressing the human and the P. vivax DHS enzymes is presented as a tool to screen for DHS selective inhibitors, the direct quantification of the two yeast expressed DHS proteins by specific antibodies is a critical baseline parameter to a reliable measure of level and stability of the target transgenic enzymes.*

We thank reviewer #1 for the comments. We hope our point-by-point answers will address your concerns.

It is true that the expression levels of the two different DHS are likely to be different; however, the amount of protein is not directly relevant for the outcome of the assay, but the enzymatic activities towards their respective target in the cellular environment. The specific activities (enzymatic activity per unit weight) of heterologous proteins from different species acting in a heterologous environment will differ, so knowing only the amount of protein expressed does not advance our understanding of the system.

These differences in DHS enzymatic activity level between the strains are taken into account by normalizing for the growth rate in the absence of external small molecules.

*2. Setting the system baseline at the yeast culture conditions which produce similar levels of eIF5A hypusination in the two DHS producing strain - i.e. 130 microM Methionine (human DHS) vs no Methionine (Pv DHS) (Met is needed to repress the yeast MET3 transcription factor driving transgene DHS expression) may not correspond to achieving production of similar amounts of the two DHSs. In absence of a direct quantification, the possible presence of different enzyme levels may bias the quantification of respective inhibition and the assessment of selectivity.*

The functional complementation is given by the amount at which the enzymes are capable of performing/replacing the endogenous enzyme function and our best available tool to measure that is by evaluating the amount of reaction product obtained. The total amount of DHS is not directly related to the activity level of the enzyme (see response to item 1 above). We believe that directly quantifying the product rather than the DHS levels is the better way to avoid bias in this case.

In addition, it should be noted that higher transcription from the *MET3* promoter (lower methionine concentration) driving expression of the respective DHS gene is required for the *P. vivax* DHS than for the human counterpart, to achieve a given growth rate. This is because genetic complementation of the absent yeast DHS by the human DHS works better than the *P. vivax* DHS. This is logical as the evolutionary distance from *Plasmodium* to human is greater than from yeast to human. Similar outcomes has been observed in analogous experimental setups, *e.g.* Bilsland *et al.* PLoS Negl Trop Dis *5*:e1320, 2011; Bilsland *et al* Open Biol 3:120158, 2013.

Thus, if we express human DHS at 130 µM methionine but need to express *P. vivax* DHS at 0 µM methionine to achieve the same growth, this is because the *Plasmodium* DHS protein complements *S. cerevisiae* DHS less well, and we need more of it to achieve the same growth. Consequently, in the strain expressing *P. vivax* DHS, the small molecule inhibitors encounter more target protein molecules. If they, despite this, are able to inhibit growth of *P. vivax* DHS strain at a lower concentration than the *H. sapiens* DHS strain, that means they have a higher affinity for *P. vivax* DHS than for *H. sapiens* DHS. So in this respect, we err on the safe side.

*3. Also, as the system is based on measuring growth complementation of the yeast strains in which yDHS is replaced by either the human or the parasite DHS enzymes, the kinetics and extent of lethality induced by the addition of Methionine is important to evaluate reliability and comparability of the system phenotipic readout.*

First, the maximum methionine concentration used in this study, 130 µM, is the same concentration as in standard synthetic complete (SC) yeast growth media with amino acids. Hence, the methionine in itself in the absence of a gene ectopically regulated by the methionine concentration, has no negative impact on growth or viability of yeast.

It should also be noted that lethality is not measured in our assay, but inhibition of growth.

We tested inhibition of cell proliferation using methionine concentrations ranging from 0 to 130 µM in the human DHS complemented strain and all allowed growth of the strain. For the strain complemented by PvDHS, concentrations above 7.5 µM completely inhibited growth (now shown in new Fig S5B) and were consequently not used in the screen.

*Reviewer #2:*

*1. The author's methods are sufficient for repeating the study and the methods used are appropriate to address the hypothesis. However, Western Blotting is not robust especially with a sample size of 3. The author's used a student's T-test for statistical consideration, but did not mention the distribution of their data. The authors should run two more blots for a total n of 5 and use a Shapiro-Wilk test to determine data distribution. The authors may then accurately assess which statistical test to consider.*

We thank reviewer #2 for the comments and suggestions.

We agree that all methods have their weaknesses; however western blotting is the best semi-quantitative option when the available tools are antibodies specific against the modified and unmodified version of a protein (D’Agostino *et al.* Front Chem 8:609942, 2020), such as in this case. Since western blot separates based on size, it also allows discrimination of the modification level of different protein species.

Regarding statistical analyses, first, we performed an F-test to assess the variance of the population. Using this variance, we then performed a t-test assuming equivalent or different variances. This is now clarified in the Methods section, “Evaluation of hypusination levels by western blot assay”.

*Reviewer #3: -Yes the objectives of the study clearly articulate the testible hypothesis mentioned.*

*-Yes the study desig is appropriate*

*-Yes the stastical analysis is corretly applied.*

*-No concern about the ethical and regulatory requirments.*

We thank Reviewer #3 for the comments and suggestions to our manuscript.

*Reviewer #4: I would like to congratulate the authors for this brilliant research they have conducted. They have designed an experimental target-based platform in yeast for screening P. vivax deoxyhypusine synthase inhibitors. This is the only protein known to contain the amino acid hypusine essential for cell viability in eukaryotes. Inhibiting DHS is a promising strategy to develop new therapeutic alternatives.*

We thank Reviewer #4 for the thoughtful suggestions to our manuscript. We bring point-by-point answers to your comments and concerns.

*No DHS inhibitors selective for parasite orthologs has previously been reported; They*

*-Genetically modified saccharomyces cerevisiae strains expressing DHS genes from Homo sapiens (HsDHS) or P. vivax DHS (Pv DHS) in place of the Wild type endogenous gene from S. cerevisiae.*

*They used inhibitors to assess the expression levels of these DHS genes. N2 exhibited dose-dependent inhibition of the PvDHS-expressing strain, achieving a substantial 95 % reduction in growth at 200 µM, 65 % at 100 µM, 30 % at 50 µM, and 13 % at 25 µM of the compound. In contrast, this compound did not significantly affect the growth of HsDHS (p > 0.05). Furthermore, N2 at 200 µM only led to a modest 17 % reduction in the growth of the wt strain, expressing yeast DHS. The selective growth reduction in the strain expressing PvDHS indicates that DHS is a major target of this compound.*

*Question: The optimization and validation of this screening platform needs to be well defined with data to validate it.*

*1. Why did the authors decide to stop at 7,5 uM for the PvDHS? The growth curve above this concentration is not shown and it would have been very important to consider other confounding factors of the parasite life stages.*

After testing several concentrations of methionine, we found that the PvDHS strain did not grow in methionine concentrations higher than 7.5 µM (now shown in new Fig S5B). This information has been emphasized on page 10 “Validation of the yeast experimental platform for DHS inhibitor identification” section.

*2. Will the DHS enzyme receptor have an effect on all the P. vivax life stages?*

In Kaiser *et al*., Bioinorg Med Chem 15:6200, 2007, it was shown that transcription of eIF5A can be observed in different developmental stages of the parasite, prominently in trophozoites. DHS transcription occurs in developmental stages suggesting its necessity in cell proliferation.

*3. In cases of mixed infections of P. falciparium, P vivax etc will the model still be valid? All of these were to be shown in the optimization data to generate concrete and impactful conclusions.*

This is beyond the scope of this manuscript. We have used simplified systems to study the effect of candidate inhibitors: a heterologous system in yeast, cultured human cells, and intraerythrocytic *P. falciparum* cultured *in vitro*. An infection is another and much more complex matter, not to mention a mixed *P. falciparum* / *P. vivax* infection.

However, the proteins of *P. vivax* and *P. falciparum* are sufficiently similar that drugs directed against a target in one of the parasites will often be active also against the corresponding target protein in the other.

**Results**

*Reviewer #1:*

*1. The baseline complementation efficiencies of the two transgenic enzymes (in 0 Met) are fairly similar (40% and 60% of the parasite vs the human DHS). In this respect, the result that a 20fold higher Met concentration is required to repress the human vs the parasite enzyme should be discussed, raising questions on the actual levels of the different DHS produced in the two strains. Also, in the experiment testing the GC7 inhibitor on the human DHS strain, inspection of the control lanes (no GC7) shows that the intensity of the band of hypusinated eIF5A at 0 Met is virtually identical to that obtained at 130 microM Met (Figure 2B). The fact that, in contrast, the viability of the strain in the two conditions is very different (Figure 2A, no GC7 curves) questions the proposed link between yeast viability and levels of hypusilation.*

The system builds on several parameters, where the dependency of one on another is not necessarily linear, and where the responsivity varies between different intervals. Thus, the transcription rate from the *MET3* promoter decreases with increasing methionine concentration, but the response is not linear, and above a certain concentration (about 100 µM) there is no further decrease (Mao *et al.* Curr Microbiol 45:37-40, 2002). Then, the turnover rates and translational efficiencies of the mRNAs (encoding PvDHS or HsDHS) driven by *MET3* promoter are unknown and can differ. The impact on growth rate of decreasing the DHS concentration or activity will vary; if the DHS activity is limiting it will have a big impact on growth, but when high it is no longer limiting, and the growth rate will be unaffected.

We acknowledge that as a limitation of our technique and that hypusine levels alone do not fully account for the differences in cell growth. We have corrected the text and have added to the Discussion: “There are limitations to our system, in that the readout proxy (cell proliferation) is only indirectly linked to the primary event (eIF5A hypusination). The dose-response relationship is therefore non-linear, and so the method is semi-quantitative. We can only use it in limited ranges of cell growth phase, inhibitor concentration, eIF5A hypusination etc. However, the method can be used to rank candidate inhibitors for their selectivity for the target protein parasite ortholog.”

Regarding Fig. 2, the hypusinated band with HsDHS at 0 µM methionine is a little stronger than at 130 µM, furthermore we must consider the ratio of hypusinated to total eIF5A. We have quantified the western blot data from Fig. 2B, and our analysis shows that the difference between relative hypusination levels without methionine and with 130 µM methionine differed by approximately 17 %. We have revised Fig. 2B providing a shorter exposure where the differences are clearer. We also include a file for inspection by the reviewers, with the other two western blots used for quantification of Fig. 2A and a table with the corresponding data.

*Reviewer #2:*

*1. All figures are of sufficient quality. Assuming appropriate statistics were used (pending the data distribution test), the authors did well with graphical representation of all figures. I am slightly concerned with figure 5, as the relative abundance does not look correct. Specifically, the wild type PB1 bands appear identical in chemiluminescence, especially when normalizing to the loading control, compared to NT and N7 treatments. Is this a fault of the representative blot? Could the authors please supply the other two blots used in a supplementary file?*

The N7 and PB1 hypusine bands are indeed similar and the quantification considering only the hypusine band is fairly similar, however we must consider the ratio between hypusinated eIF5A / total eIF5A. This is in turn calculated after normalizing for the amount of eEF2 (loading control) on the respective blot; thus a ratio of four numbers. We have less total eIF5A for both PB1 and N2, in comparison with NT and N7; and when we divided normalized hypusine by normalized total eIF5A and calculate the ratio, the relative hypusination is superior for N2 and PB1 compounds.

Still, considering your remark, we revised our quantification using shorter exposures, and the numbers are now corrected. The hypusination is still increased for those compounds, but less, and the differences are not statistically significant.

We also include a file for inspection by the reviewers, with the other two western blots used for quantification of Fig. 5A and a table with the corresponding data.

*Reviewer #3: -Yes the analysis match the analysis plan*

*-Yes tghe results are clearly and completely presented*

*-yes the quality of the figurs is the same.*

*Reviewer #4: The results are clearly presented and stem from the methods used.*

*1. However, as a question is raised in the methodology, the results are equally affected. Optimization results, and what happens in case of mixed infection.*

We hope to have addressed your concerns about that in our previous response to your questions.

--------------------

**Conclusions**

*Reviewer #1: The conclusion that the described two yeast strain system is ready to screen for selective inhibitors of Plasmodium vivax DHS is overstated. The characterisation of the baseline features of the biological system requires further experiments.*

With this system we improved limitations such as increased sensibility to external compounds, consequently reducing the amount of compound needed to be used in an HTS campaign. We have now discussed about the limitations of our platform characterization in terms of quantification of hypusination, growth phase, and complementation efficiency.

*Reviewer #2: The authors provide a short and sweet discussion summarizing their findings and discussing their model's future use. This is sufficient and appropriate.*

*Reviewer #3: Yes the conclusion support the data.*

*Reviewer #4: The conclusion is good and direct to the point based on the objective of the study carried out.*

--------------------

**Editorial and Data Presentation Modifications**

*Reviewer #1: page 2:*

*1. “Shortage of in vitro culture methods” is incorrect and too generic for the Plasmodium genus, whereas it is appropriate for P. vivax.*

We modified the sentence to clarify: “While continuous culture of *P. falciparum* in its intraerythrocytic stage is possible, there is currently shortage of continuous *in vitro* culturing system **for *P. vivax***; experimentation thus requires constant replenishment of parasites from patient samples.”

*2. page 3: Essentiality of DHS is overemphasized and incorrectly quoted. In reference to the pathogens (refs 14-16), an essential role is demonstrated only in the case of Trypanosome brucei; in P. falciparum, growth defects associated to the induced decreased amounts of PfDHS led to speculate of essentiality; finally, ref 16 does not contain experiments showing an essential role of DHS in the parasite studied there.*

The previous reference 16 has been removed and new references giving ample evidence of the

essential role of DHS in *Plasmodium* spp, *Leishmania donovani* and in *Fusarium*

*graminearum* cell proliferation by gene knockout or inhibition are now provided: Bushell et

al. Cell, 170:260-272, 2017; Zhang *et al.* Science 360:eaap7847, 2018; Kersting *et al.* FEBS

Open Bio, **6**:860-72 2016; Chawla *et al.* J Biol Chem, 285:453-463, 2010; Woriedh *et al.*

Mol Plant Microbe Interact, 24:619-627, 2011.

*3. page 4: The new strain is “approx 60-fold more sensitive to the human DHS inhibitor GC7” compared to? (the parental line, presumably).*

We modified the sentence to clarify: “~60-fold more sensitive to the human DHS inhibitor GC7, compared to a human DHS-complemented strain lacking these genome editions previously generated by us (Silva *et al*. PLoS Negl Trop Dis. 14:e0008762, 2020)”

*4. page 5: It is suggested to describe the key features of the parasite specific a.a. insertions in PvDHS compared to the yeast and the human DHSs: number, size, location in the primary sequence.*

We added the suggested description to page 5: “The enzyme core of PvDHS shares many features with its human homologue. In fact, the PvDHS protein sequence exhibits 59 % similarity with the human DHS and 60 % with *S. cerevisiae* DHS (Fig. 1). Moreover, the residues in both the GC7 binding site and the allosteric site are conserved between HsDHS, ScDHS and PvDHS (as illustrated in Fig. 1). However, PvDHS differs from the human and yeast DHS in their overall length, PvDHS has a total extension of 455 amino acids, being 86 amino acids longer than HsDHS and 68 longer than ScDHS. Notably, these unique insertions are mainly located from the N-terminal to the middle of the protein extension (Fig. 1). The larger insertion (residues 224 to 263) aligns with residues that form an alpha helix in the human enzyme (α8 in Fig. 1). Another small insertion, located from residues 100 to 107, aligns with α3 in the human DHS. Currently, it remains unclear whether or how these extensions impact the PvDHS protein.”

*5. page 9: It may be useful to mention the rationale for engineering the strain with two different fluorescent reporters; the use is incidentally mentioned in reference to the robotic screening.*

It is now described as it follows: “Engineering the strains with fluorescent tags enables growth competition assays for different parasite targets with higher precision than using turbidity as the readout (Alalam *et al.* mSystems 6:e01087, 2021) allowing simultaneous evaluation of parasite target against the human equivalent within the same well (Bilsland *et al.* Open Biol, 3:120158, 2013).

*6. pages 10-11: See main comments above on the baseline features of the biological system.*

See our replies to Reviewer #1 Methods item 1 and Results item 1.

*7. page 14: The description of hypusine as “the final product of the reaction catalysed by DHS” is slightly misleading; the DHS-DOOX two step pathway to produce hypusin-eIF5a was more accurately described in the Introduction.*

The sentence is now adjusted: “decrease in the final product (hypusine) of the reaction which DHS catalyzes the first step…” We corrected the information every time we used it along the text to avoid misleading. Thank you for the remark.

*8. pag 15 – Figure 5: The remarkable increase in the level of yeast eIF5a hypusination induced by treatment with the inhibitors N2 and PB1 deserves attention and discussion.*

A new version of the Figure with revised quantification is now provided. We previously used parts of the western blot that where the signal was saturated. Now, using shorter exposures of the same blots, we are within the linear response range and can get better estimates.

*9. pag 16 – Discussion: Analysis and comments on the structure of the identified hits is expected in this section. In the case of N2 and N7, in comparison with those of the described human enzyme competitive and the allosteric inhibitors. In the case of PB1, which shows some structural similarity with the allosteric inhibitor of the human DHS, a comparison with the structural binding studies proposing its anti-tubercolosis activity could be worth mentioning.*

We included discussion about the compounds:

“Our molecular docking studies show that compound N2 binds the predicted orthosteric site of *P. vivax*, while N7 binds to the allosteric site. With a similar ligand interaction profile as GC7, N2 forms hydrogen bonds with the amino acids Gly395A, Glu404A and Lys410A, as well as a hydrophobic interaction with Trp408A. Additionally, unlike GC7, N2 exhibits a halogen bond with Asn192B via the chlorine atom. Predicted interactions for N7 include a halogen bond between the fluorine atom and Phe310A, a hydrogen bond with the side chain of Lys410A and multiple hydrophobic interactions, such as with Trp128A and Ile359A. No protein targets had been previously reported for these compounds.

PB1 was identified through the Pathogen box screening rather than molecular docking. This compound was previously told to have dual inhibitory activity *in vitro* against the *Mycobacterium tuberculosis* proteins EthR and InhA (Mugumbate *et al.* Front. Pharmacol 8:681, 2017). We now propose *P. vivax* DHS as a novel target for this small molecule.”

*Reviewer #2: (No Response)*

*Reviewer #3: Minor Revision with explanation*

*Reviewer #4: -*

--------------------

**Summary and General Comments**

*Reviewer #1: The manuscript describes an interesting in vivo system using two S. cerevisiae strains respectively engineered with a human and a malaria parasite (P. vivax) enzyme, DHS, which catalyses the first step of a reaction leading to a unique conserved aminoacid modification, the hypusination of EIF5a. Proposing to ultimately identify selective parasite DHS inhibitors, the manuscript presents the use of the human DHS competitive inhibitor GC7 to validate the yeast system and aims to use the failure to complement the deleted yeast DHS in the two strains as a way to screen for selective inhibitors of the parasite DHS. The conclusion that the system is ready to screen for selective inhibitors of P. vivax DHS is however overstated in the absence of the characterisation of key baseline features of the biological system.*

Thank you for this assessment. We hope that we have clarified all the points raised by this reviewer in the Methods, Results, and Conclusions sections. The main strength of our system is the ability to identify small molecule inhibitors that are selective for a parasite target protein over its human ortholog. We acknowledge that there are limitations to our system, in that the readout proxy (cell proliferation) is only indirectly linked to the primary event (eIF5A hypusination). The dose-response relationship is therefore non-linear, and so the method is only semi-quantitative. We can only use it in limited ranges of cell growth phase, inhibitor concentration, eIF5A hypusination etc. However, the method can be used to rank candidate inhibitors for their selectivity for the target protein parasite ortholog.

*Reviewer #2: Overall, the authors have completed what they set out to do. Figure 5 aside, they have done a great job in establishing a baseline yeast model for screening P. vivax drug targets. Given the lack of long-term in vitro culturing techniques available for vivax, this model is novel and has high impact potential.*

*Reviewer #3: Authors have tried to identify inhibitors of DHS from Plasmodium vivax and used genetically modified Saccharomyces cerevisiae strains expressing DHS genes from Homo sapiens (HsDHS) or P. vivax (PvDHS) in place of the endogenous DHS gene from S. cerevisiae. The manuscript is interesting but requires some explanation. Authors are suggested to respond to the comments below and resubmit the manuscript.*

1. *Paragraph 2 of abstract: “This new strain background was -----" which new strain is authors referring to?*

We modified the sentence to clarify: “Compared to a HsDHS complemented strain with a different genetic background previously generated by the author, this new strain background was ~60‑fold more sensitive to an inhibitor of human DHS than the one previously used.” (see response to Reviewer #1, Editorial, item 3).

2. *Why authors have not proposed the same experiments on P. falciparum as the invitro culture is easy?*

We want to focus on *P. vivax* first because of the shortage of drugs directed at this parasite. On the other hand, the reviewer is right to point out that it will be entirely feasible to target the *P. falciparum* DHS also with the system we describe here.

*3. Result 3: Establishment of S. cerevisiae……Antibody details of ScDHS missing.*

The reference for the antibody anti-Dys1 is now added to the following sentence in the section: “…The deletion of ScDHS was confirmed by western blot, as no band was detected with the anti-Dys1 antibody (Galvao *et al.* *PLoS One*, 8:e60140, 2013) for *S. cerevisiae* DHS...”

*4. Why were the newly developed strains not checked for expression of Hs DHS and Pv DHS in the newly developed strains are specific antibodies available for the same?*

Currently there are no specific antibodies available for PvDHS, so the characterization would be incomplete. We tested if the antibody anti-HsDHS would cross react with PvDHS but it did not.

*5. Discussion should be detailed out more*

We have now added more detail to the Discussion, as suggested. For instance, we have explained how we adjusted methionine concentrations to achieve comparable eIF5A hypusination levels when expressing human or parasite DHS, we have elaborated the predicted contacts between the inhibitors and *P. vivax* DHS, and clarified other items requested by the reviewers.

*Reviewer #4: Authors should address the worries raised in the methodology*

See our responses to Reviewer #1 Methods item 1, Reviewer #2 item 1 and Reviewer #4 items 1, 2, and 3.

--------------------
